# Agarose Hydrogels Enriched by Humic Acids as the Complexation Agent

**DOI:** 10.3390/polym12030687

**Published:** 2020-03-19

**Authors:** Martina Klučáková

**Affiliations:** Faculty of Chemistry, Brno University of Technology, Purkyňova 118/464, 612 00 Brno, Czech Republic; klucakova@fch.vutbr.cz; Tel.: +42-054-114-9410

**Keywords:** hydrogel, agarose, humic acid, reactivity, diffusion

## Abstract

The transport properties of agarose hydrogels enriched by humic acids were studied. Methylene blue, rhodamine 6G and Cu(II) ions were incorporated into hydrogel as diffusion probes, and then their release into water was monitored. Cu(II) ions as well as both the dyes studied in this work have high affinity to humic substances and their interactions strongly affected their diffusion in hydrogels. It was confirmed that humic acids retarded the transport of diffusion probes. Humic acids’ enrichment caused the decrease in the values of effective diffusion coefficients due to their complexation with diffusion probes. In general, the diffusion of dyes was more affected by the complexation with humic acids in comparison with Cu(II) ions. The effect of complexation was selective for the particular diffusion probe. The strongest effect was obtained for the diffusion of methylene blue. It was assumed that metal ions interacted preferentially with acidic functional groups. In contrast to Cu(II) ions, dyes can interact with acidic functional groups, and the condensed cyclic structures of the dye probes supported their interactions with the hydrophobic domains of humic substances.

## 1. Introduction

Hydrogels play an important role in the monitoring of the mobility of pollutants in nature as well as in their removal and water treatment. They are usually based on the materials able to absorb water and different pollutants in their structure. There are many studies and reviews dealing with bio-polymeric and polymeric hydrogels for different environmental applications. Agarose, pectin, alginate or lignin may be mentioned as examples of materials eligible for these purposes [1,2,3,4,5,6,7,8,9]. This work is focused on the agarose and its hydrogels as a medium for the investigation of their transport properties and effect of humic substances on the release of metal ions and dyes from them. Agarose hydrogels have a number of practical utilizations. They can be, e.g., used as separation media in column chromatography and act as bacterial culture support [1,2]. Their gelling [3], rheological and thermal properties [7,8,9], and internal structure [10,11,12] have been widely studied, but the effect of micro-scale structural factors of porous media on effective mass diffusion is not well understood [11], and the molecular structure of agarose is still a matter of debate [3,7]. Some studies stated that the hydrogels consist of thick bundles of agarose chains and large pores of water [10,13], which constitute the main paths for diffusing particles. The hydrogel is widely used as a transport medium for the determination of diffusion characteristics of different molecules and ions [2,13,14,15,16]. Pluen et al. [14] studied the diffusion of several different macromolecules through 2% agarose hydrogel. Data were analysed by means of Yimm-Rouse model [17] and the reptation model [13,18]. Gong et al. [13] investigated the effect of aspect ratio of protein on its diffusion in hydrogel and the effect of electrostatic interaction between protein and hydrogel on its transport through hydrogel. The influence of of electrostatic and specific interactions on the diffusion and partitioning of various solutes in agarose hydrogels was studied by Fation-Rouge et al. [2]. Gutenwik et al. [15] determined diffusion coefficients of proteins at different pH values and ionic strengths by means of diffusion cells. Golmohamadi et al. [16] measured the self and mutual diffusion of different cations and correlated them with Donnan potentials of hydrogels. Wang et al. [19] characterized the diffusion of cations and anions in thin films of agarose hydrogel. Agarose hydrogel is often used as transport medium passive samplers based on diffusion gradients in thin hydrogel films [19,20].

As can be seen, agarose hydrogels are widely studied and utilized as transport media for different diffusing particles. In our previous studies [21,22,23,24], agarose hydrogels were enriched by humic acids as an active component in order to support the complexation of diffusing particles in hydrogels. Humic-agarose hydrogels were characterized by means of their viscoelastic properties [21,24], and the transport of different ions through the hydrogels was studied by means of diffusion cells [21,23,24] and non-stationary transient diffusion [22,23]. The effect of acidic functional groups of humic acids on the complexation and transport of metal and dye ions was investigated by means of the selective blocking of carboxylic groups by methylation [23,24]. Our experiments showed that the addition of humic acids in agarose hydrogel can strongly influence the complexation and diffusion of metal ions and dyes, which resulted in changes in effective diffusion coefficients.

In this study, a different type of experiment was performed. The study is focused on transport properties of agarose hydrogels enriched with humic acids, especially the release of different diffusion probes from the hydrogels. Agarose hydrogel was enriched by humic acids and also by metal or dye ions. The transport out of hydrogel was monitored and the diffusion characteristics of the transport were determined. Simultaneously, the degree of immobilization of ions was calculated, and the ratio between mobile and complexed ions was calculated. The aim was to investigate the influence of interactions of humic acids with probes on their release ability.

## 2. Experimental

### 2.1. Materials

Agarose (AG; routine use class), CuCl_2_·2H_2_O (p.a.), methylene blue hydrate (MB; CI basic blue 9) and rhodamine 6G (RH; CI basic red 1) were purchased from Sigma-Aldrich (St. Luis, MO, USA).

Samples of humic acids were purchased from the International Humic Substances Society (IHSS, St. Paul, MN, USA). Elliot soil humic acids (ESHA), Pahokee peat humic acids (PPHA), Suwannee river humic acids (SRHA) and Leonardite humic acids (LEHA) were used in this study. The main characteristics such as elemental composition and the contents and properties of acidic functional groups can be found on the website of the International Humic Substances Society (IHSS).

### 2.2. Preparation of Hydrogels

The preparation of hydrogels was based on the thermo-reversible gelation of AG aqueous solution. An accurately weighed amount of AG was dissolved in deionized water or in an aqueous solution of humic acids. The mixture was slowly heated with continuous stirring up to 80 °C and stirred at this temperature in order to obtain a transparent solution, and finally sonicated (1 min) to remove gasses. AG hydrogels were prepared using 1 wt % AG solution [21,22,23,24]. Afterwards, the AG solution was slowly poured into the polymethylmethacrylate (PMMA) spectrophotometric cuvette (inner dimensions: 10 mm × 10 mm × 45 mm). The cuvette orifice was immediately covered with pre-heated plate of glass to prevent drying and shrinking of gel. Flat surface of the boundary of resulting hydrogels was provided by wiping an excess solution away. Gentle cooling of cuvettes at the laboratory temperature led to the gradual gelation of the mixture [22,23]. AG–HA hydrogels were prepared from 1 wt % AG solution containing 0.01 wt % of HA. The AG and humic contents in final hydrogels were chosen on the basis of our previous results and experimental experiences [21,22,23,24]. Images of pure agarose hydrogel and hydrogel enriched by humic acids in cuvettes are shown in Figure S1 (from the Supplementary Materials).

Aqueous solutions of CuCl_2_, MB, and RH were used as the donor solutions for the incorporation of the diffusion probes into hydrogels. Their initial concentrations were equal to 0.1 mol.dm^−3^ for Cu(II) salt and 1 mg.dm^−3^ for dyes. The incorporation of the probes into hydrogels was based on diffusing of Cu(II) ions and dyes into the hydrogels. The cuvettes filled with hydrogels were placed into stirred donor solutions (4 cuvettes in 200 cm^3^). The diffusion probes have been diffusing into the hydrogel until a constant concentration throughout the whole hydrogel was achieved [25,26]. 

### 2.3. Diffusion-Release Experiments

The cuvettes with AG and AG–HA hydrogels enriched by diffusion probes were placed in stirred distilled water (4 cuvettes in 200 cm^3^). The release of diffusion probes into water was monitored over time. The concentrations of probes in leachates were measured by means of UV-VIS spectrometer Hitachi U3900H (Hitachi, Tokyo, Japan). The data were used for the calculation of diffusion fluxes from the hydrogels into water through the square orifices of the cuvettes. 

Simultaneously, the distributions of diffusion probes in hydrogels were determined in selected time intervals. The cuvettes were taken out of the leachates and the UV-VIS spectra were measured at various distances from the orifice by means of Varian Cary 50 UV-VIS spectrophotometer equipped with the special accessory providing controlled fine vertical movement of the cuvette in the spectrophotometer. Using the collected UV-VIS spectra, the concentrations of the probes were determined at different positions in gels [22]. The obtained data were used to compute the concentration profiles of probes in the cuvettes. The diffusion fluxes determined as the differences between the total contents of probes in hydrogels before diffusion experiments and the contents in hydrogels at given times should be the same as the values calculated on the basis of the concentrations measured in leachates; therefore their values were determined by two different measurements and averaged. All experiments were performed at laboratory temperature (25 ± 1 °C). Data are presented as average values with standard deviation bars. Schematic illustration of release experiment is shown in Figure S2 (from the Supplementary Materials).

## 3. Results and Discussion

In this work, the effect of standard humic acids as the complexation agents added in agarose hydrogels was studied by means of so-called diffusion-release experiments. Humic acids are known as substances which complex effectively with metal ions [25,26,27,28,29,30,31,32,33] and dyes [21,22,23,24]. Carboxylic functional groups, as well as aromatic structures and π−π interactions, are important in their reactivity. The amounts of diffusion probes in hydrogels differed slightly according to type of added humic acids (Table 1). 

The contents differed more in the case of organic dyes. Their amounts in hydrogels without humic acids seem to be higher in comparison with enriched hydrogels. In contrast, the content of copper is slightly higher. It is well known that humic acids have very high affinity to Cu(II) ions [24,25,26,27,28,30,31,32,33]. Therefore, copper is a traditional model metal used to study humic reactivity. The increase in the content of Cu(II) ions in agarose hydrogels enriched by humic acids can be considered as the result of this humic affinity observed also in our previous studies [24,25,26,27,28]. If we compare the contents of Cu(II) ions in hydrogels containing different humic acids with the contents of their acidic functional groups declared by IHSS [34,35], we can find that the content of Cu(II) ions in hydrogels increases with the increasing total acidity of studied humic acids. This confirmed that the acidic functional groups play the most important role in the interactions of humic acids with metal ions. Nevertheless, we must take account of the strengths (dissociation abilities) of functional groups and the fact that metal ions can be bound by other active centres, as studied in detail in [27]. It should be noted that IHSS published the content of acidic functional groups related to the content of carbon in humic acids and it is necessary to re-calculate the data on the whole samples of humic acids. On the other hand, this increase is not high, which means that only a smaller portion of metal present in hydrogel can be bonded by humic acids which corresponds with the low content of humic acids in hydrogel. No relationship exists between content of dyes in hydrogels and amounts of functional groups. There are more possibilities for the binding of dyes by humic acids. Apart from dissociable functional groups, the unsaturated and aromatic structures are more asserted due to the aromatic structure of studied dyes. 

The knowledge of contents of diffusion probes was necessary for the mathematical description of their release from hydrogels. The effective diffusion coefficients *D*_ef,h_ of Cu(II) ions and dyes in hydrogels were calculated on the basis of the following equation [26,36,37]:

(1) $$m_{h\to s}=2\frac{\varepsilon c_{0,h}-c_{0,s}}{1+\varepsilon \sqrt{D_{s}/D_{\mathrm{ef},h}}}\sqrt{\frac{D_{s}t}{\pi }}$$

where *m*_h__→__s_ is the total diffusion flux at time *t*; *c*_0,h_ and *c*_0,s_ are the initial concentrations of the probe in the hydrogel and aqueous solution (equal to zero in this case); *D*_ef,h_ and *D*_s_ are the effective diffusion coefficient of the probe in the hydrogel and the diffusion coefficient of the probe in the supernatant; *ε* is the ratio between concentrations of the probe in the supernatant (*c*_s_) and hydrogel (*c*_h_) in given time, i.e., *ε* = *c*_s_/*c*_h_. 

The values of *D*_s_ for Cu(II) ions are tabulated [38]: 1.43 × 10^−9^ m^2^·s^−1^. The values of *D*_s_ for dyes were determined in our previous study [22]. They were extrapolated for 25 °C and used in this work as: 8.42 × 10^−10^ m^2^·s^−1^for MB and 8.93 × 10^−10^ m^2^·s^−1^for RH [24]. These results are in agreement with values determined using other methods [16,19,39,40,41]. 

**Figure 1:**
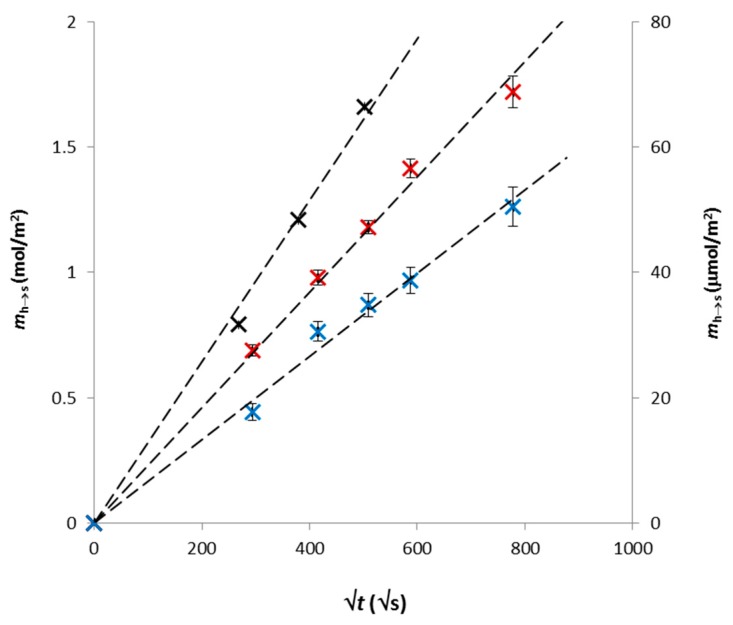
Experimental data obtained for Cu (**black**), MB (**blue**) and RH (**red**) fitted by Equation (1).

Experimental data fitted by Equation (1) are shown in Figure 1. We can see that they are in good agreement with the mathematical model. The slopes of the lines were used for the calculation of effective diffusion coefficients *D*_ef,h_. Their values are listed in Table 2.

The highest values of effective diffusion coefficients were determined for pure AG hydrogel. The obtained values can be compared with the results published in other studies [16,19,40,42,43,44]. Wang et al. [19] characterized the agarose hydrogel used in so-called DGT technique (diffusive gradients in thin films) for monitoring of different substances in natural environments (waters, soils, sediments). They investigated diffusivities of several ions including Cu(II) in 1.5 wt % agarose hydrogel and determined the value diffusion coefficient equal to 6.59 × 10^−10^ m^2^·s^−1^. The value obtain in other study [43] for 2% agarose hydrogel was slightly lower (6.59 × 10^−10^ m^2^·s^−1^). In this study, practically double value of *D*_ef,h_ = 1.22 × 10^−9^ m^2^·s^−1^. This difference is partially caused by lower content agarose in our hydrogel and partially by different methods used for the determination of diffusivity, which is principal for the resulting value of the diffusivity [44]. The published values of diffusion coefficients of MB in 1.5 wt % agarose hydrogel (enriched by 3 wt % CaCl_2_) were between 2.9 and 3.9 × 10^−10^ m^2^·s^−1^ depending on the MB concentration and pH [40]. Similarly, the diffusion coefficients of RH in in 1.5 wt % agarose hydrogel were between 2 and 3.5 × 10^−10^ m^2^·s^−1^ depending on the concentration and pH [16]. Variations in obtained values of diffusion coefficients of RH were observed also for its diffusion in water (2.8–4.3 × 10^−10^ m^2^·s^−1^) [44].

In the case of release of Cu(II) ions, the highest value of *D*_ef_ was determined for the AG–LEHA hydrogel. The same hydrogel achieved the highest initial content of Cu(II) ions. The LEHA sample can be characterized by the highest total acidity, and C/H and C/N ratios; aromaticity; and the lowest O/C ratio [34]. The lowest value of *D*_ef_ was determined for the AG–ESHA hydrogel which can be characterized by the lowest C/N ratio, but C/H and O/C are comparable with LEHA. Simultaneously, ESHA has relatively high total acidity and aromaticity. The mobility of dyes in AG–ESHA and AG–LEHA hydrogels were similar. Both humic acids have high C/H and low O/C ratios. They are also more aromatic in comparison with other two samples. In contrast, the highest diffusivity of MB in the AG-PPHA hydrogel is probably caused by common impact of low content of acidic functional groups, C/H and C/N ratios and low aromaticity.

Chakraborty et al. [42] combined DGT with the CLE (competing ligand exchange) technique in order to investigate diffusion of metal ions in the presence of humic substances. They observed the increase in the diffusion coefficients from 6.06 × 10^−10^ m^2^·s^−1^ (obtained for Cu(II) ion) to 6.2 × 10^−11^ m^2^·s^−1^ (obtained for Cu–NLHA complex), 8.0 × 10^−11^ m^2^·s^−1^ (obtained for Cu–NLFA complex), and 8.5 × 10^−11^ m^2^·s^−1^ (obtained for complex of Cu with Suwannee River natural organic matter). Similarly, the decrease in diffusion coefficient of Cu–HA in comparison with free Cu(II) ions in the hydrogel based on polyacrylamide cross-linked with an agarose derivative was from 5.48 × 10^−10^ to 5.70 × 10^−11^ m^2^·s^−1^ [43]. In contrast, the effect of humic acids on the diffusion of RH in water was much weaker: from 2.88 × 10^−10^ to 2.22 × 10^−10^ m^2^·s^−1^ for RH-PPHA complex and 2.15 × 10^−10^ m^2^·s^−1^ for RH-SRHA complex. Their values of diffusion coefficient in 1.35 wt % AG hydrogels achieved 91%, 87% and 88% of diffusion coefficients in water, respectively [5]. It means that the effect of humic substances on the diffusion in agarose hydrogels observed by different authors differed. This finding showed that we must be very careful in the comparison of diffusion characteristics obtained by different authors and different methods [44].

The decrease of effective diffusion coefficients obtained for the hydrogels enriched by humic acids can be the result of two effects. The first is a possible change in hydrogel structure (see SEM of lyophilized hydrogels in Figure S3, from the Supplementary Materials). In spite of the fact that the content of humic acids in hydrogel is relatively low, their incorporation into AG hydrogel can influence its inner structure, including the distribution, size and shape of hydrogel pores [24]. The structure of humic acids is very dynamic and sensitive to circumstances such as concentration, pH and ionic strength [24,45,46]. They can be characterized by a supramolecular arrangement of relatively small particles in co-existence with bigger macromolecules [24,45,46,47,48,49,50,51], which makes it possible to respond to changes in their surroundings. 

The second effect is a possible interaction between the diffusion probes and HA and humic acids during the transport of the probes through the hydrogel. In comparison with our previous works [21,22,23,24,25,26,27,28], the diffusion probes are in equilibrium with humic acids at the beginning of the release experiments. It means that the immobilization of the probe has the same rate as its liberation from binding sites. It is known that diffusion probes occurring in the hydrogels can be divided into three fractions: free mobile particles without chemical binding to humic acids, ion exchangeable ions bound by electrostatic forces, and strongly (covalently) bound particles in humic complexes [25,28,52]. These fractions are in a dynamic equilibrium and can convert to other forms as a result of changes in circumstances. 

**Figure 2:**
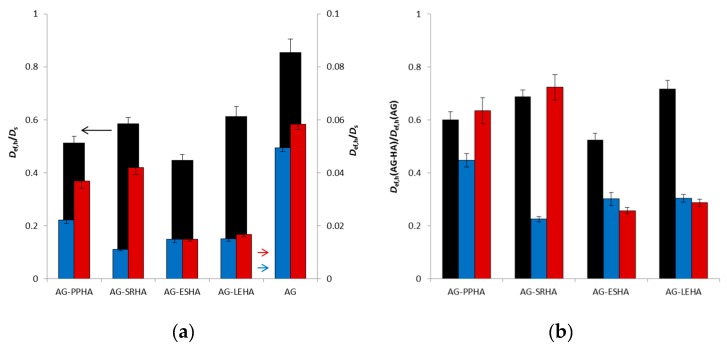
The ratios between effective diffusion coefficients *D*_ef,h_ and the diffusion coefficients of probes in aqueous solutions *D*_s_ (**a**); the ratios between effective diffusion coefficients *D*_ef,h_ for AG-HA hydrogels and the values obtained for pure AG hydrogel (**b**): Cu (black); MB (blue); and RH (red).

In the case of release experiments, the mobile fraction can easily diffuse out of the hydrogel. It results in the displacement from equilibrium, and particles of probe can be liberated from the exchangeable and strongly bound fractions. These processes can strongly affect the release of probe from AG–HA hydrogels and are dependent on the character of humic acids. In Figure 2, the ratios between effective diffusion coefficients *D*_ef,h_ and the diffusion coefficients of probes in aqueous solutions *D*_s_ and the ratios between effective diffusion coefficients *D*_ef,h_ for AG–HA hydrogels and the values obtained for pure AG hydrogel are shown. The differences between dyes and Cu(II) ions were observed. While Cu(II) ions amount to 40%–80% of their diffusion coefficients in solution, that proportion is only 1%–6% in the case of dyes, mainly because of their sizes. The liberation of dyes from their ion-exchangeable and strongly bound fractions has an influence comparable with Cu(II) ions (AG–PPHA and AG–SRHA) or lower (AG–ESHA and AG–LEHA). The values of *D*_ef,h_ obtained for hydrogels enriched by humic acids amount to 20%–70% of the values obtained for pure AG hydrogel.

The influence of inner structure of hydrogel on the diffusion can be characterized as so-called structure fraction *μ*, which is the ratio between the porosity *ϕ* and tortuosity *τ*:

(2) μ=ϕ/τ

The value of *μ* can be determined as the ratio between the diffusion coefficient of the probe in AG hydrogel and the diffusion coefficient of the probe in water (*D*_s_). If we focus on the ratios obtained for pure AG hydrogel, we can state that their values are lower for the diffusion of dyes (5%–6%) in comparison with Cu(II) ions (> 80%). In the case of AG–HA hydrogels, the situation is more complex. The release of diffusion probes from hydrogels can be described as the non-stationary diffusion based on Fick’s equation [36,37]:

(3) $$\frac{\partial c}{\partial t}=D_{\mathrm{ef},h}\frac{\partial^{2}c}{\partial x^{2}}$$

where *c* represents the concentration of the diffusing compound at time *t* and position *x* (the coordinate parallel to the direction of the diffusion movement). The diffusion coefficient *D*_ef,h_ is the main parameter characterizing the rate of the transport. The diffusion coefficient is an “effective” characteristic which reflects the influence of chemical interactions of diffusion probe with humic acids in their transport through the hydrogel and the influence of inner structure of hydrogel. Mathematically, the effects of the chemical reaction can be described by the following equation based on the conservation of mass:

(4) $$\frac{\partial c}{\partial t}=D^{*}\frac{\partial^{2}c}{\partial x^{2}}-\dot{r}$$

where D^*^ is the diffusion coefficient affected only by the porous structure of the hydrogel and $\dot{r}$ is the rate of chemical reaction. In this case, the value of D^*^ is equal to *D*_ef,h_ for pure AG hydrogel. If a fast chemical reaction in the presence of local equilibrium between free mobile probes (*c*) and immobilized ones (*c*_im_) is presumed (*K* is the equilibrium constant), then

(5) $$c_{\mathrm{im}}=Kc$$

and Equation (4) can be written as

(6) $$\frac{\partial c}{\partial t}=D^{*}\frac{\partial^{2}c}{\partial x^{2}}-K\frac{\partial c}{\partial t},$$

and consequently,

(7) $$\frac{\partial c}{\partial t}=\frac{D^{*}}{1+K}\frac{\partial^{2}c}{\partial x^{2}}=D_{\mathrm{ef},h}\frac{\partial^{2}c}{\partial x^{2}}$$

Since the diffusion coefficient in the hydrogel D^*^ is dependent on its porosity and tortuosity expressed by the structural factor *μ* according to the Equation (2), the following relation can be written:

(8) $$D_{\mathrm{ef},h}=\frac{D^{*}}{1+K}=\frac{\mu D_{s}}{1+K}$$

in which the effects of the tortuous movement of the diffusing matter in the hydrogel and the chemical reaction between diffusion probe and humic acids are involved [25,26,27,28,36,37].

The values of *K* can be calculated only assuming that the inner structure of hydrogel was not changed by the addition of humic acids and they should be proportional to the ratios between effective diffusion coefficients *D*_ef,h_ for AG–HA hydrogels and the values obtained for pure AG hydrogel shown in Figure 2b. As it was described in [24], rheological measurements showed that the AG hydrogel is more resistant to applied stress than hydrogels enriched with humic substances and the networks of the AG–HA hydrogels can easily collapse. The behaviour of hydrogels enriched with humic substances shifted towards that of viscoelastic liquids. This means that hydrogels containing humic substances had a lower ability to resist mechanical stresses, which can be connected with their higher permeability. Therefore the effect of interactions between diffusion probes and humic acids is probably higher than the values of *K* listed in Table 3.

**Figure 3:**
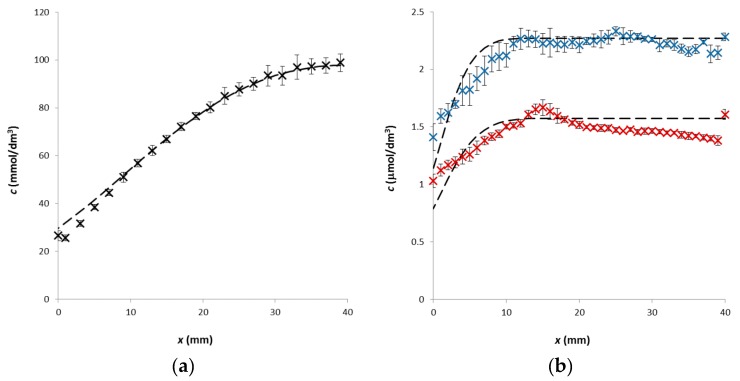
The concentration profile of Cu(II) ions in AG–LEHA hydrogel after 40 h from the start of release (**a**), and the concentration profiles of MB (blue) and RH (red) in AG–LEHA hydrogel after 168 h from the start of release (**b**) fitted by Equation (8) – dashed curves.

The concentration profile in hydrogel during the release of Cu(II) ions and dyes can be described as [25,36,37]:

(9) $$c=\frac{1}{2}c_{0,h}\left[erf\frac{l-x}{2\sqrt{D_{\mathrm{ef},h}t}}+erf\frac{l+x}{2\sqrt{D_{\mathrm{ef},h}t}}\right]$$

where *l* is the length of hydrogel and *x* is the distance from the interface between hydrogel and solution. This model is in a good agreement with data obtained for Cu(II) ions (see Figure 3). The small differences were observed close to the interface between hydrogels and solutions. The agreement between mathematical model and experimental data showed on the fact that the release of Cu(II) ions corresponded with our presumptions and they were accumulated on the interface. Similar results were obtained for all studied AG–HA hydrogels and Cu(II) ions. In contrast, the agreement of the Equation (8) with experimental data obtained for dyes was worse. It seems that dyes are accumulated in a certain distance from the interface. This was observed mainly in the case of RH (see Figure 3b). It is not easy to explain it. It is known that MB and RH can form bigger aggregates [53,54,55,56,57]. This formation together with general bigger size of dye probes (in comparison with metal ions) can support the observed accumulation.

As mentioned above, the transport through hydrogel can be affected by two factors: the tortuous movement of the diffusing particles in the porous structure of hydrogel and the interactions of diffusing particles with hydrogel. On the condition that pure agarose hydrogel cannot interact with a diffusion probe, we can determine the influence of the porous structure on the diffusion. The decrease in the diffusivity of probes in pure agarose hydrogels (in comparison with the diffusivity in water) can be attributed fully to the tortuosity effect. In general the movement of diffusing particles can be suppressed by their sizes. Particles of dyes are generally bigger than metal ions; therefore their Brownian motion is less intensive. On the other hand, pore size of agarose hydrogel exceed significantly the Stokes hydrodynamic radius of dyes [21,58]. The decrease is much stronger for dyes which can be connected with their sizes. The accumulation of dyes in a certain distance from the interface is more likely connected with the humic acids contained in enriched hydrogel. No accumulation was observed in the case of diffusion in pure agarose hydrogel. The most intensive accumulation was observed for the hydrogel enriched by LEHA, the weakest for ESHA. It is not easy to explain this finding. The phenomenon was observed only in release experiments. It means that dye was homogeneously distributed in hydrogel and (partially) complexed with humic acids and the equilibrium between humic acids, dye and formed complexes in the beginning is assumed. When the release of dye from hydrogel started, the equilibrium was distorted and some dye can be liberated from humic complexes. We assumed that the observed accumulation can be connected with the disruption of equilibrium and an effort of the system to attain new equilibrium. It seems that humic-dye complexes are (partially) able to diffuse towards the interface between hydrogel and water, but their movement is much slower in comparison with free dye particles. It resulted in the situation wherein an excess of free dye particles arose in the hydrogel closer to interface and their depletion in the hydrogel far from the interface; therefore, the equilibrium must be attained again and again as the release proceeds. This means that a part of free movable dyes can be complexed in the hydrogel closer to interface and a part of complexes can be disintegrated in the hydrogel far from the interface. Other effect is that the pores in hydrogel are filled by solution containing both free dyes and probably also by their complexes with humic acids which can obstruct the movement of smaller free dye particles. Both these effects probably resulted in the described state and the maximum observed on the concentration profile of dye in hydrogel. Different types of humic acids (extracted from different matrices) were used in order to compare their abilities to interact with diffusion probes and influence their release out of hydrogel. It is necessary to realize further experiments in order to investigate our findings in detail. 

## 4. Conclusions

The influence of humic acids on the transport of metal ions and dyes in agarose hydrogel was studied. It was confirmed that humic acids retarded the transport of diffusion probes. Humic acids’ enrichment caused decreases in the values of effective diffusion coefficients due to their complexation with diffusion probes. The effect of complexation was selective for the particular diffusion probe. The strongest effect was obtained for the diffusion of MB in the AG–SRHA hydrogel, the lowest one for the diffusion of Cu(II) ions in the AG–PPHA hydrogel. In general, the diffusion of dyes was more affected by the complexation with humic acids in comparison with metal ions. We assume that metal ions interacted preferentially with acidic functional groups. In contrast, dye can interact with acidic functional groups and the condensed cyclic structure of the dye probes supported their interactions with the hydrophobic domains of humic substances.

The results can be used in the investigation of the functioning of natural organic matter in the transport of pollutants in natural systems. Humic acids, as important constituents of soil organic matter, are able to affect, significantly, the migration and bioavailability of some pollutants in nature. In this study, agarose hydrogel was used as a model of a system with a homogeneous distribution of humic acids contaminated by metal ions and dyes. This model hydrogel was very wet in order to study the release of pollutants out of hydrogel. The purpose was to assess the effect of humic acids (as the constituent of soil organic matter) on the mobility of pollutants in wet soil. It means that pollutants present in soil can be partially complexed by humic substances and the movements of complexed and free pollutants are generally restricted if the soil is dry. In contrast, pollutants can diffuse relatively fast in wet soils, and their movement can be supported also by a convection in soggy soils. This study was focused on the diffusion of pollutants in a model of wet soil (e.g., after rain). Results described in this study showed that some pollutants complexed by humic acids are (partially) able to diffuse through pore structure, but their movement is slower and can cause an accumulation of dyes in a certain position (distance from interface). This accumulation can influence the ensuing release from the pore structure into water (e.g., it can reduce the effective size of pores). Therefore, the results obtained in this study can help in the investigation of the functioning of natural organic matter in the transport of pollutants in natural systems. The effective diffusion coefficient determined on the basis of this study included the influence of pore structure and the interactions between humic substances and pollutants. Both pores and affinity of organic matter to pollutants differ with the type and quality of soil. Therefore, the methods and technics presented in this study can be used for predictions of the mobility and bioavailability of pollutants. The mathematical models for different diffusion processes are “universal” and can be used for different hydrogel materials (inert and reactive) and different diffusion probes. One of them could be the use of humic hydrogels as the material having controlled release of nutrients in agriculture.

## Figures and Tables

**Table 1:** Concentrations of diffusion probes in the AG and AG–HA hydrogels before diffusion-release experiments.

Hydrogel	Cu: *c*_0,h_ (mmol.dm^−3^)	MB: *c*_0,h_ (mmol.dm^−3^)	RH: *c*_0,h_ (mmol.dm^−3^)
AG	85.3 ± 7.1	7.6 ± 0.4	6.4 ± 0.1
AG–ESHA	91.2 ± 7.6	6.9 ± 0.6	6.0 ± 0.2
AG–PPHA	90.8 ± 5.9	5.7 ± 0.3	4.6 ± 0.1
AG–SRHA	88.6 ± 6.6	5.2 ± 0.2	4.4 ± 0.2
AG–LEHA	96.2 ± 8.2	7.3 ± 0.2	5.7 ± 0.1

**Table 2:** The values of effective diffusion coefficients of diffusion probes in the AG and AG–HA hydrogels.

Hydrogel	Cu: *D*_ef,h_ (10^−10^ m^2^·s^−1^)	MB: *D*_ef,h_ (10^−11^ m^2^·s^-1^)	RH: *D*_ef,h_ (10^−11^ m^2^·s^−1^)
AG	12.21 ± 0.72	4.16 ± 0.13	5.21 ± 0.16
AG–ESHA	6.40 ± 0.30	1.25 ± 0.10	1.33 ± 0.06
AG–PPHA	7.34 ± 0.36	1.86 ± 0.21	3.30 ± 0.25
AG–SRHA	8.39 ± 0.30	0.93 ± 0.04	3.76 ± 0.25
AG–LEHA	8.76 ± 0.38	1.26 ± 0.06	1.49 ± 0.07

**Table 3:** Values of the apparent equilibrium constants *K* determined on the basis of Equation (8).

Hydrogel	Cu: *K* (-)	MB: *K* (-)	RH: *K* (-)
AG–ESHA	0.64 ± 0.03	2.32 ± 0.19	2.90 ± 0.13
AG–PPHA	0.22 ± 0.01	1.23 ± 0.07	0.58 ± 0.04
AG–SRHA	0.37 ± 0.01	3.45 ± 0.15	0.38 ± 0.02
AG–LEHA	0.89 ± 0.01	2.29 ± 0.11	2.49 ± 0.12

## Supplementary Materials

The following are available online at https://www.mdpi.com/2073-4360/12/3/687/s1, Figure S1: Pure agarose hydrogel (left) and agarose hydrogel enriched by humic acids (right) in cuvettes, Figure S2: Schematic illustration of release experiment, Figure S3. SEM of lyophilized hydrogels (ZEISS EVO LS 10): pure agarose hydrogel (left) and agarose hydrogel enriched by humic acids (right).
