# Supplementary material for: Agarose Hydrogels Enriched by Humic Acids as the Complexation Agent
Source: Polymers (Basel). 2020 Mar 19;12(3):687. doi: 10.3390/polym12030687 (PMC7182926; doi:10.3390/polym12030687)
Supplement: Supplementary file 1 [file polymers-12-00687-s001.pdf]

Supplementary Materials

Agarose Hydrogels Enriched by Humic Acids as the Complexation Agent

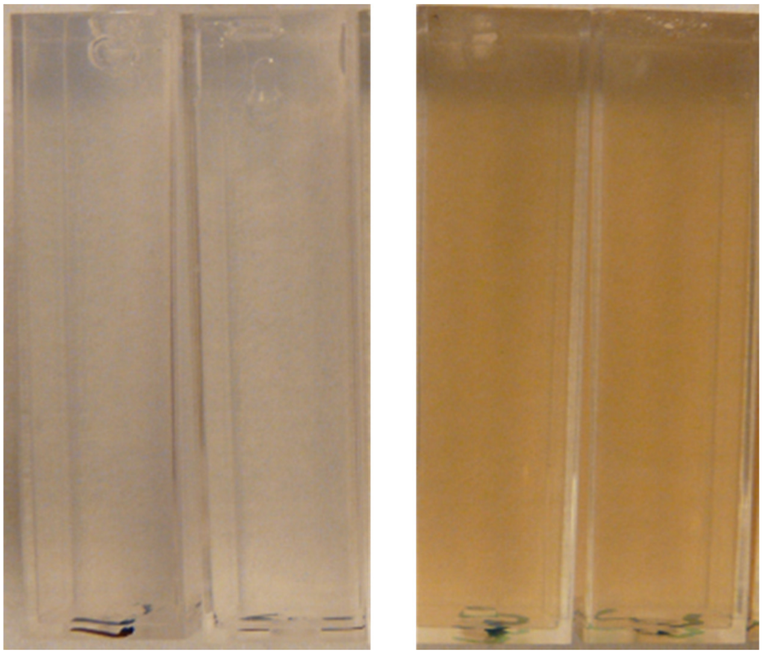

Figure S1. Pure agarose hydrogel (**left**) and agarose hydrogel enriched by humic acids (**right**) in cuvettes.

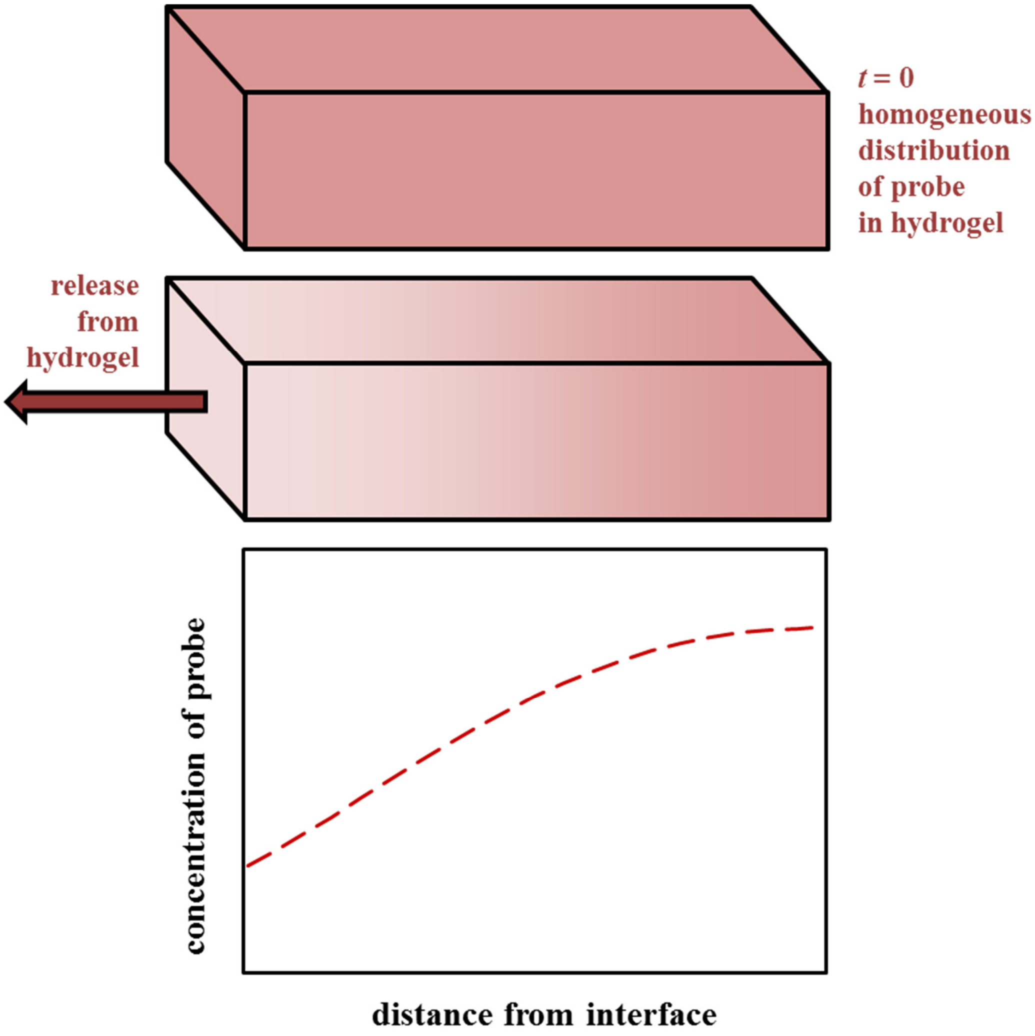

Figure S2. Schematic illustration of release experiment

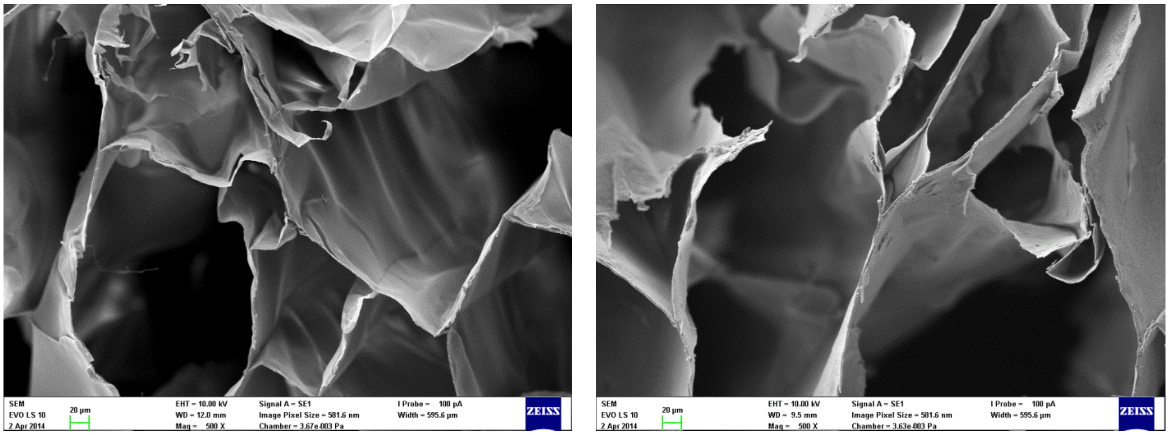

Figure S3. SEM of lyophilized hydrogels (ZEISS EVO LS 10): pure agarose hydrogel (**left**) and agarose hydrogel enriched by humic acids (**right**)
